# Glycosides from the leaves of *Fraxinus Hubeiensis*

**DOI:** 10.1186/s13065-023-01070-6

**Published:** 2023-12-13

**Authors:** Xin-Yi Liu, Hong-Xia Tang, Wen-Bing Sheng, Qu-Jing Luo, Lin-Xi Mao, Yu-Pei Yang, Xiao-Zhou Guo, Qing-Lai Wu, Yu-Qing Jian, Wei Wang, Xu-Dong Zhou

**Affiliations:** 1 TCM and Ethnomedicine Innovation & Development International Laboratory, School of Pharmacy, Hunan University of Chinese Medicine, 410208 Changsha, People’s Republic of China; 2 Institute of Pesticides, School of Agriculture, Yangtze University, 434020 Jingzhou, People’s Republic of China

**Keywords:** *Fraxinus hubeiensis*, 3-keto-glycoside, Spectroscopic analysis, α-glucosidase inhibitory, Chiral separation

## Abstract

*Fraxinus hubeiensis* is a plant endemic to China and widely used as folk medicine to treat various diseases. However, its chemical constituents have never been reported sufficiently. Thus, the primary objective of this study was to investigate the phytochemical constituents and biological activities of *F. hubeiensis* leaves. Hence, combined column chromatographic and spectroscopic techniques were used to identify and characterize the secondary metabolites such as a pair of 3-keto-glycoside epimers (**1**) and (**2**), along with five known compounds (**3** ~ **7**). The results of α-glucosidase inhibitory activity exhibited that **1** and **2** had moderate activity with IC_50_ values of 359.50 and 468.43 µM, respectively, compared to a positive control acarbose with the IC_50_ value of 164.08 µM. However, Compounds **1**–**6** were shown to be inactive against the tested microbes.

**Supplementary Information:**

The online version contains supplementary material available at 10.1186/s13065-023-01070-6.

## Introduction

*Fraxinus hubeiensis* belongs to the family *Oleaceae* which comprises about 27 genera and more than 400 species in the world [1, 2]. About 30 species of the *Fraxinus* genus native to China and some of which were used in traditional Chinese Medicines as Cortex Fraxini [3], commonly known as ‘Qin-Pi’ in Chinese. Because of its very narrow distribution and relatively long growth cycle, *F*. *hubeiensis*, as a rare species, has received little attention [4]. In folk applications, it has also been widely used in bonsai due to its exquisite shape. However, the phytochemical study for *F*. *hubeiensis* has barely been reported [5–7], which showed that five coumarins, one flavonoid, one steroid, one iridoid glycosides and two fatty acids have been found from the leaves and barks of *F. hubeiensis* [5] to the best of our knowledge. Besides, the previous research was mainly focused on the essential oil and its antimicrobial effects. In continuing our search for diversity of phytochemistry and bioactivities from *F*. *hubeiensis* [5], we have investigated the constituents of the leaves of this plant, which led to the isolation of seven compounds, including a pair of 3-keto-glycoside epimers ethyl *α*-D-*ribo*-hex-3-ulopyranoside (**1**) and ethyl *β*-D-*ribo*-hex-3-ulopyranoside (**2**), as well as five known compounds (**3**–**7**). In this paper, we described the isolation and structural elucidation of these compounds by spectroscopic analysis and comparison with those of literature values. The α-glucosidase inhibitory activity of compounds **1** and **2** were evaluated. Meanwhile, compounds **1**–**6** were screened for antimicrobial activity. Thus, all of these could lay the foundation for discussing the chemotaxonomic research on *Fraxinus* and elucidating its active ingredients, and indicate great potential for the development of novel plant-derived hypoglycemic drugs.

## Results and discussion

**Fig. 1:**
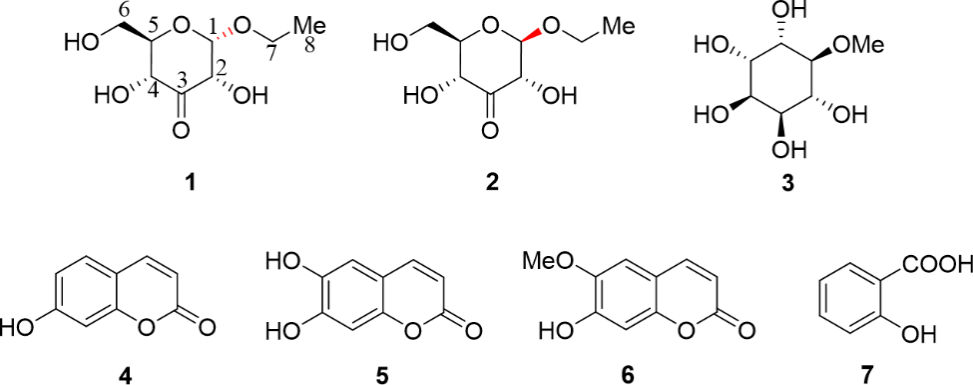
Chemical structures of **1–7** isolated from *F. hubeiensis*

The ethyl acetate (EtOAc) extract (189.0 g, 3.78% yield) form a 5.0 kg sample of *F. hubeiensis* was loaded to column chromatography (CC) on silica gel to yield eight fractions, which were purified further by repeated CC and semi-preparative HPLC to afford a mixture of **1** (1.5 mg) and **2** (2.1 mg), **3** (5.3 mg), **4** (4.0 mg), **5** (7.0 mg), **6** (5.2 mg) and **7** (21.4 mg) (Fig. 1).

Initially, the mixture (**1** and **2**) was obtained and exhibited a single spot in TLC and one peak detected over a rountine HPLC equipment. Its ^1^ H and ^13^ C NMR spectra showed two sets of resonances respectively, and can be assigned clearly to each compound, which suggested the mixture was a racemic. The above deduction was in accordance with its optical activity which was close to zero. To confirm, subsequent chiral HPLC of the mixture resulted in the purification of two enantiomers in a ratio of 1:2, with opposite optical rotation values.

**Fig. 2:**
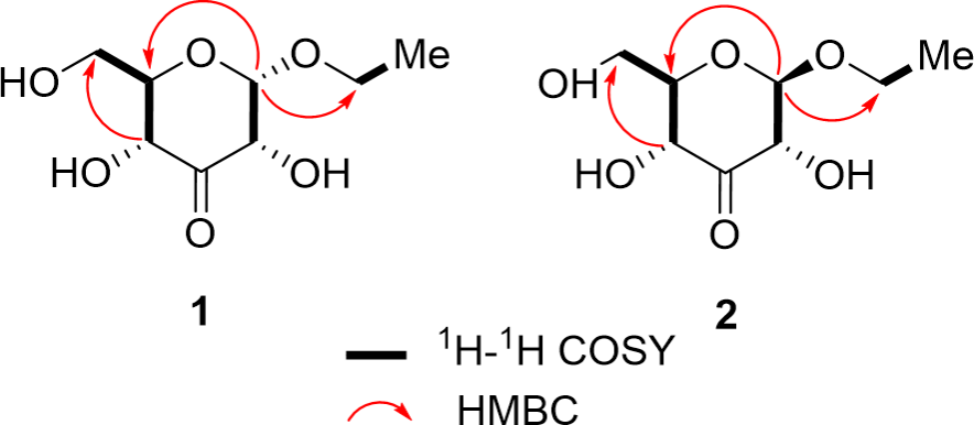
Key ^1^ H-^1^ H COSY and HMBC correlations of compounds **1** and **2**

The molecular formula of *α*-D-*ribo*-hex-3-ulopyranoside (**1**) was determined as C_8_H_14_O_6_ based on its positive HR-ESI-MS data of [M + Na]^+^ peak at *m/z* 229.0683 (calcd for 229.0688) and supported by the ^13^ C-NMR data (Table 1), indicating two degrees of unsaturation. The IR spectrum of **1** exhibited the presence of hydroxy at 3512 cm^-1^, carbonyl at 1652 cm^-1^. The ^1^ H-NMR data of **1** exhibited the presence of one methyl group resonating at *δ*_H_ 1.20 (t, *J* = 7.1 Hz); two methylene groups at *δ*_H_ 3.80 (dd, *J* = 12.0, 4.7 Hz, H-6a), 3.86 (d, *J* = 12.0, 2.2 Hz, H-6b), and 3.54 (dq, *J* = 9.8, 7.1 Hz, H-9a), 3.78 (dq, *J* = 9.8, 7.1 Hz, H-9b); four methine group at *δ*_H_ 5.16 (d, *J* = 4.3 Hz, H-1), 4.39 (dd, *J* = 4.3, 1.6 Hz, H-2), 4.22 (dd, *J* = 9.7, 1.6 Hz, H-4) and 3.69 (ddd, *J* = 9.7, 4.7, 2.2 Hz, H-5), combined with its DEPT and HSQC spectrum. All eight carbons were well resolved in the ^13^ C NMR spectrum, which indicated the presence of a carbonyl carbon in the downfield region. The connectivity of CH (4)- CH (5)-CH_2_ (6) could be well deduced from the cross-peaks of H-4/H-5/H-6 in the ^1^ H-^1^ H COSY spectrum (Fig. 2). Meanwhile, the linkage of CH (1)-CH (2) was established based on the cross-peaks of H-1/H-2. The detailed analysis of 1D and 2D NMR spectra implied **1** had a 3-keto-glycoside skeleton, along with their comparison with those of the reported glycoside derivatives. The remaining ethoxy group based on the peak shapes from the ^1^ H-NMR and ^1^ H-^1^ H COSY spectrum, was linked with C-1 to form the glycoside, considering the molecular formula and the unsaturation degrees. For the stereochemistry, the anomeric proton exhibited the relatively small coupling constant (*J* = 4.3 Hz), supporting the *α*-glycosidic configuration. Thus, the structure of **1** was elucidated as *α*-D-*ribo*-hex-3-ulopyranoside as shown in Fig. 1.

**Table 1:** The ^1^ H NMR (600 MHz) and ^13^ C NMR (151 MHz) data of **1** (in CD_3_OD) and **2** (in CD_3_OD) (*δ *in ppm, *J* in Hz)

No.	1		2	
	*δ*_C_	*δ*_H_	*δ*_C_	*δ*_H_
1	102.5	5.16, d (4.3)	105.6	4.37, d (7.9)
2	76.0	4.39, dd (4.3, 1.6)	78.2	4.11, dd (7.9, 1.8)
3	207.1		207.1	
4	73.6	4.22, dd (9.7, 1.6)	73.7	4.22, dd (10.1, 1.8)
5	76.8	3.69, ddd (9.7, 4.7, 2.2)	78.3	3.31, overlapped
6	62.5	3.80, dd (12.0, 4.7)	62.6	3.79, dd (12.1, 4.9)
		3.86, dd (12.0, 2.2)		3.93, dd (12.1, 2.1)
7	64.9	3.54, dq (9.8, 7.1)	66.5	3.68, dq (9.6, 7.1)
		3.78, dq (9.8, 7.1)		4.01, dq (9.6, 7.1)
8	15.1	1.20, t (7.1)	15.4	1.26, t (7.1)

*β*-D-*ribo*-hex-3-ulopyranoside (**2**), was also obtained as colorless gum. The molecular formula was determined to be C_8_H_14_O_6_ based by HR-ESI-MS peak at [M + Na]^+^ peak at *m/z* 229.0697 (calcd for 229.0688). Interesting that, the ^1^ H and ^13^ C-NMR signals were different from those of its enantiomer **1**, even when they were mixed together. Meanwhile, the HMBC cross-peaks confirmed the structure of **2** (Fig. 2), which was previously found as a biotransformation product in the *Coleus forskohlii* root culture induced from *Agrobacterium rhizogenes* [8]. The large coupling constant (*J* = 7.9 Hz) of the anomeric proton indicated it had *β*-glycosidic configuration. Therefore, the structure of **2**, as a new natural product, was determined as *β*-D-*ribo*-hex-3-ulopyranoside.

The known compounds (Fig. 1) were characterized as methylinsitol (**3**) [9], umbelliferone (**4**) [10], esculetin (**5**) [11], scopoletin (**6**) [12], salicylic acid (**7**) [13], by comparison of their NMR data with those of the reported literature.

The α-glucosidase inhibition assay was carried out for a pair of 3-keto-glycoside epimers **1** and **2**. Compounds **1** and **2** exhibited moderate activities with their IC_50_ values of 359.50 and 468.43µM respectively, compared to acarbose with the IC_50_ value of 164.08µM (Table 2). Meanwhile, the antimicrobial activities of compounds **1**–**6** were tested, while none of them exhibited activities.

**Table 2:** Inhibitory effects of compounds **1** and **2** on α-glucosidase

Compounds	IC_50_(µM)
**1**	359.50±21.72
**2**	468.43±4.43
Acarbose^a^	164.08±17.29

^a^Acarbose was used as a positive control

## Experimental

### General experimental procedures

Optical rotations were measured on an Autopol-III automatic polarimeter (Rudolph Research Analytical, USA). IR spectra were recorded on an Agilent Technologies Cary 630 FTIR spectrophotometer with KBr pellets. NMR spectra were acquired on Bruker AV-600 spectrometer using methanol-d_4_ (CD_3_OD) or chloroform-d_1_ (CDCl_3_) as references, locked to the deuterium signal of the solvent. Chemical shifts were given in parts per million (ppm), and coupling constants in hertz (Hz). HRESIMS were measured on a Waters UHPLC-H-CLASS/XEVO G2-XS Q-TOF mass spectrometer (Waters, USA). Semi-preparative HPLC was performed using an Agilent 1260 Infinity II liquid chromatograph system equipped with UV-VIS detector. And a reversed-phase C18 column (Agilent Eclipse XDB, 250 × 9.4 mm., 5 μm) and a chiral-phase column (NQ (2)-RH, 250 × 4.6 mm, 5 μm) were used for purification and enantioselective analysis. Thin-layer chromatography (TLC) was conducted using silica gel GF_254_ (Qingdao Haiyang Chemical Co. Ltd.) plates. Open column chromatography was performed using silica gel (Qingdao Haiyang Chemical Co. Ltd., 200–300 mesh) and Sephadex LH-20 (Pharmacia Biotech Ltd.) and reverse-phase C_18_ silica gel ODS (Merck & Co., Inc. USA). All the solvents were of analytical grade and were purchased from Beijing Chemical Company Ltd. TLC spots were observed under UV light or by spraying with 5% sulfuric acid vanillin solution.

α-glucosidase and acarbose from *Saccharomyces cerevisiae*, p-nitrophenyl-α-glucopyranoside (pNPG) were obtained from Sigma-Aldrich Co. (St. Louis, MO, USA), and they were all stored at − 20 °C before using. *Staphylococcus aureus* [*S. aureus*, CMCC (B) 26,003], was purchased from Shanghai Luwei Technology Co., Ltd.). *Escherichia coli* (*E. coli*, ATCC 25,922), *Pseudomonas aeruginosa* (*P. aeruginosa*, ATCC 27,853) and *Candida albicans* [*C. albicans*, CMCC (F) 98,001] and *Bacillus subtilis* (*B. subtilis*), were all purchased from Shanghai Bioresource Collection Center.

### Plant materials

The dried leaves of *F*. *hubeiensis* were collected in the Bonsai garden of Jingzhou in China, in June 2017. Plant materials were identified by one of the authors (Prof. Qing-Lai Wu). A voucher specimen (No. DJBL201706) was deposited at TCM and Ethnomedicine Innovation & Development International Laboratory in Hunan University of Chinese Medicine.

### Extraction and isolation

The dried leaves of *F*. *hubeiensis* (5.0 kg) were pulverized and immersed in 40 L 95% aqueous EtOH for three times at room temperature, each time 10 days. The solvent was evaporated under vacuum to obtain a crude extract (1.01 g), and the extract was suspended in 2 L H_2_O and partitioned successively with petroleum ether (PE, 60 ~ 90 °C) and ethyl acetate (EtOAc), to yield PE (311.0 g), EtOAc (189.0 g), and water (420.1 g) fractions. The EtOAc fraction was isolated by column chromatography (CC) using silica gel (200–300 mesh) and eluted with PE/CH_2_Cl_2_ (9:1), PE/EtOAc (9:1), CH_2_Cl_2_/EtOAc (9:1), CH_2_Cl_2_/MeOH (9:1), CH_2_Cl_2_/MeOH (1:1) gradientally, to yield eight fractions (Fr.A-H).

Fr. A (2.1 g) was loaded to silica gel CC and eluted with CH_2_Cl_2_/EtOAc (1:0 ~ 0:1) to obtain twelve subfractions (Fr. A1-A12), and subfraction Fr. A9 was absorbed to CC with Sephadex LH-20, eluting with CH_2_Cl_2_/MeOH (1:1) to obtain fourteen subfractions and yield compound **4** (4.0 mg) and **6** (5.2 mg). Fr. C (5.1 g) was absorbed to silica gel CC (CH_2_Cl_2_/EtOAc,1:0 ~ 0:1) to obtain eight major subfractions (Fr.C1-C8). And then Fr.C5 was loaded to a Sephadex LH-20 CC (CH_2_Cl_2_/MeOH, 1:1) and recrystallized to yield compound **5** (7.0 mg).

Fr. G (40.3 g) was isolated by CC on silica gel, eluting with CH_2_Cl_2_/EtOAc (1:0 ~ 0:1) to get ten subfractions (Fr. G1-G10), and Fr.G9 (1.2 g) was loaded on a Sephadex LH-20 CC, eluting with MeOH to obtain eight subfractions (Fr.G9.1-G9.8). Fr. G9.4 was purified on Sephadex LH-20 CC, eluting with MeOH to yield compound **3** (5.3 mg). Fr. G9.2 was purified by semi-preparative HPLC (MeOH/H_2_O, 35:65, 3 mL/min) to yield a mixture of **1** and **2** (4.0 mg, *t*_R_: 3.5 min), exhibiting a single peak, whose value of optical rotation was close to zero. Therefore, the mixture of **1** and **2** was separated on a Chiral NQ (2)-RH Column (MeOH/H_2_O, 75:25, 0.7 mL/min) to obtain **1**(1.5 mg), *t*_R1_:4.7 min and **2** (2.1 mg), *t*_R2_:5.2 min.

Compound (**1**): white, amorphous gum; $[{{\upalpha }]}_{D}^{25}$ + 5.8 (*c* 0.06, MeOH); UV (MeOH) λ_max_ (log *ε*): 198 (1.43) ,282 (0.38) nm; IR (KBr) *ν*_max_3512, 2958, 1652, 1506, 1032, 1018 cm^−1^; ^1^ H and ^13^ C NMR data (CD_3_OD, 600/151 MHz), see Table 1; HRESIMS *m/z* 229.0683 [M + Na]^+^ (calcd for C_8_H_14_O_6_Na^+^, 229.0688).

Compound (**2**): white, amorphous gum; $[{{\upalpha }]}_{D}^{25}$ – 16.0 (*c* 0.08, MeOH); UV (MeOH) λ_max_ (log *ε*): 198 (1.19), 286 (0.13) nm; IR (KBr) *ν*_max_3512, 2958, 1652, 1506, 1032, 1018 cm^− 1^; ^1^ H and ^13^ C NMR data (CD_3_OD, 600/151 MHz), see Table 1; HRESIMS *m/z* 229.0697 [M + Na]^+^ (calcd for C_8_H_14_O_6_Na^+^, 229.0688).

### α-glucosidase inhibitory assay

The α-glucosidase inhibition assay was carried out on the basis of the method reported with minor modifications [14, 15]. Briefly, 2µL of test sample (0.1, 0.25, 0.5, 1.0 mmol/L) dissolved in 98µL of PBS buffer (0.1 mol/L, pH 6.8) and 25µL α-glucosidase (0.2 U/mL) solution were mixed and pre-incubated at 37 °C for 20 min in a 96-well microplate. Then, 25µL p-nitrophenyl-α-D-glucopyranoside (pNPG, 4 mmol/L) solution was added to initiate the reaction. The 96-well microplate was incubated at 37 °C for an additional 15 min before being stopped with 50µL Na_2_CO_3_ (0.2 mol/L). The absorbance of each well was measured at a wavelength of 405 nm, and the data were recorded and measured in parallel for three times. In this experiment, acarbose was used as the positive control, PBS buffer was used as the blank group, and DMSO solution was used as the negative control group. Other reagents were consistent with the sample experimental group. The absorbance at 405 nm was measured by microplate reader, and the inhibition rate was calculated.

The α-glucosidase inhibitory activity was expressed as percent inhibition and was calculated as follows:

$$Inhibition\,\% \, = \,\left( {1 - \frac{{Ab{s_{sample}} - Ab{s_{blank}}}}{{Ab{s_{negative\,\,control}}}}} \right)*100\%$$

### Antimicrobial activity

Compounds **1**–**6** were screened for antimicrobial activities against the tested microbes (*S. aureus*, *B. subtilis*, *E. coli*, *P. aeruginosa* and *C. albicans*) by the previously reported method [16, 17]. Regrettably, compounds **1–6** exhibited no activities. Perhaps for these isolates, the rang of strains should be expanded to test for the activity. However, because of no sufficient of these compounds isolated from this plant, it was not able to screen them more broadly for antimicrobial activities.

## Conclusion

In summary, seven compounds were isolated from the leaves of *F. hubeiensis* including two ketoglycosides, one methylinsitol, three coumarins and salicylic acid. All compounds except **5** were isolated from *F. hubeiensis* for the first time. This study is the first reported of successful isolation of a pair of 3-ketoglycoside isomers by chiral-phase HPLC, which are undescribed compounds from nature. The results of α-glucosidase inhibitory indicated that **1** and **2** possessed moderate activities. It was worth mentioning that the activity of **1** with *α*-configuration was better than that of **2** with *β*-configuration, so the subsequent work on structural modification based on the ketose-type skeleton and their structure-activity relationship deserves further study. According to traditional uses of the plant, compounds **1**–**6** were evaluated for antimicrobial effect. Unfortunately, these isolates had no activity. Therefore, more in-depth phytochemistry and pharmacological research is worth continuing in the future.

### Electronic supplementary material

Below is the link to the electronic supplementary material.

Supplementary Material 1

## Data Availability

All data generated or analysed during this study are included in this current article and its supplementary information files.
