# Supplementary material for: Glycosides from the leaves of Fraxinus Hubeiensis
Source: BMC Chem. 2023 Dec 13;17(1):182. doi: 10.1186/s13065-023-01070-6 (PMC10720128; doi:10.1186/s13065-023-01070-6)
Supplement: Supplementary file 1 — Supplementary Material 1 [file 13065_2023_1070_MOESM1_ESM.docx]

**Supplementary data**

**Glycosides from the leaves of *Fraxinus hubeiensis***

Xin-Yi Liu ^a, †^, Hong-Xia Tang ^a, †^, Wen-Bin Sheng ^a^, Qu-Jing Luo ^a^, Lin-Xi Mao ^a^, Yu-Pei Yang^a^, Xiao-Zhou Guo ^a^, Qing-Lai Wu ^b^, Yu-Qing Jian ^a^, Wei Wang ^a*^, Xu-Dong Zhou ^a*^

^a^ *TCM and Ethnomedicine Innovation & Development International Laboratory, School of Pharmacy, Hunan University of Chinese Medicine, Changsha, 410208, P.R. China*

^b^ *Institute of Pesticides, School of Agriculture, Yangtze University, Jingzhou, 434020, P.R.China;*

^†^ *Xin-Yi Liu and Hong-Xia Tang contributed equally to this study.*

^*^Corresponding author. Tel: +86 731 8845 8240; fax: +86 8845 8227.

*E-mail address*: [wangwei402@hotmail.com](mailto:wangwei402@hotmail.com) (W. Wang).

^*^Corresponding author. Tel/Fax: +86 731 8845 8240

*E-mail address:* [xudongzhou999@163.com](mailto:xudongzhou999@163.com) (X.-D. Zhou).

Contents:

Fig. S1. ^1^H NMR spectrum of *α*-D-*ribo*-hex-3-ulopyranoside (**1**) (CD_3_OD, 600 MHz).

Fig. S2. ^13^C NMR spectrum of *α*-D-*ribo*-hex-3-ulopyranoside (**1**) (CD_3_OD, 151 MHz).

Fig. S3. DEPT 135°spectrum of *α*-D-*ribo*-hex-3-ulopyranoside (**1**).

Fig. S4. HSQC spectrum of *α*-D-*ribo*-hex-3-ulopyranoside (**1**).

Fig. S5. HMBC spectrum of *α*-D-*ribo*-hex-3-ulopyranoside (**1**).

Fig. S6. ^1^H-^1^H COSY spectrum of *α*-D-*ribo*-hex-3-ulopyranoside (**1**).

Fig. S7. HR-ESIMS spectrum of *α*-D-*ribo*-hex-3-ulopyranoside (**1**).

Fig. S8. UV spectrum of *α*-D-*ribo*-hex-3-ulopyranoside (**1**).

Fig. S9. IR spectrum of *α*-D-*ribo*-hex-3-ulopyranoside (**1**).

Fig. S10. ^1^H NMR spectrum of *β*-D-*ribo*-hex-3-ulopyranoside (**2**) (CD_3_OD, 600 MHz).

Fig. S11. ^13^C NMR spectrum of *β*-D-*ribo*-hex-3-ulopyranoside (**2**) (CD_3_OD, 151 MHz).

Fig. S12. DEPT 135° spectrum of *β*-D-*ribo*-hex-3-ulopyranoside (**2**).

Fig. S13. HSQC spectrum of *β*-D-*ribo*-hex-3-ulopyranoside (**2**).

Fig. S14. HMBC spectrum of *β*-D-*ribo*-hex-3-ulopyranoside (**2**).

Fig. S15. ^1^H-^1^H COSY spectrum of *β*-D-*ribo*-hex-3-ulopyranoside (**2**).

Fig. S16. HR-ESIMS spectrum of *β*-D-*ribo*-hex-3-ulopyranoside (**2**).

Fig. S17. UV spectrum of *β*-D-*ribo*-hex-3-ulopyranoside (**2**).

Fig. S18. IR spectrum of *β*-D-*ribo*-hex-3-ulopyranoside (**2**).


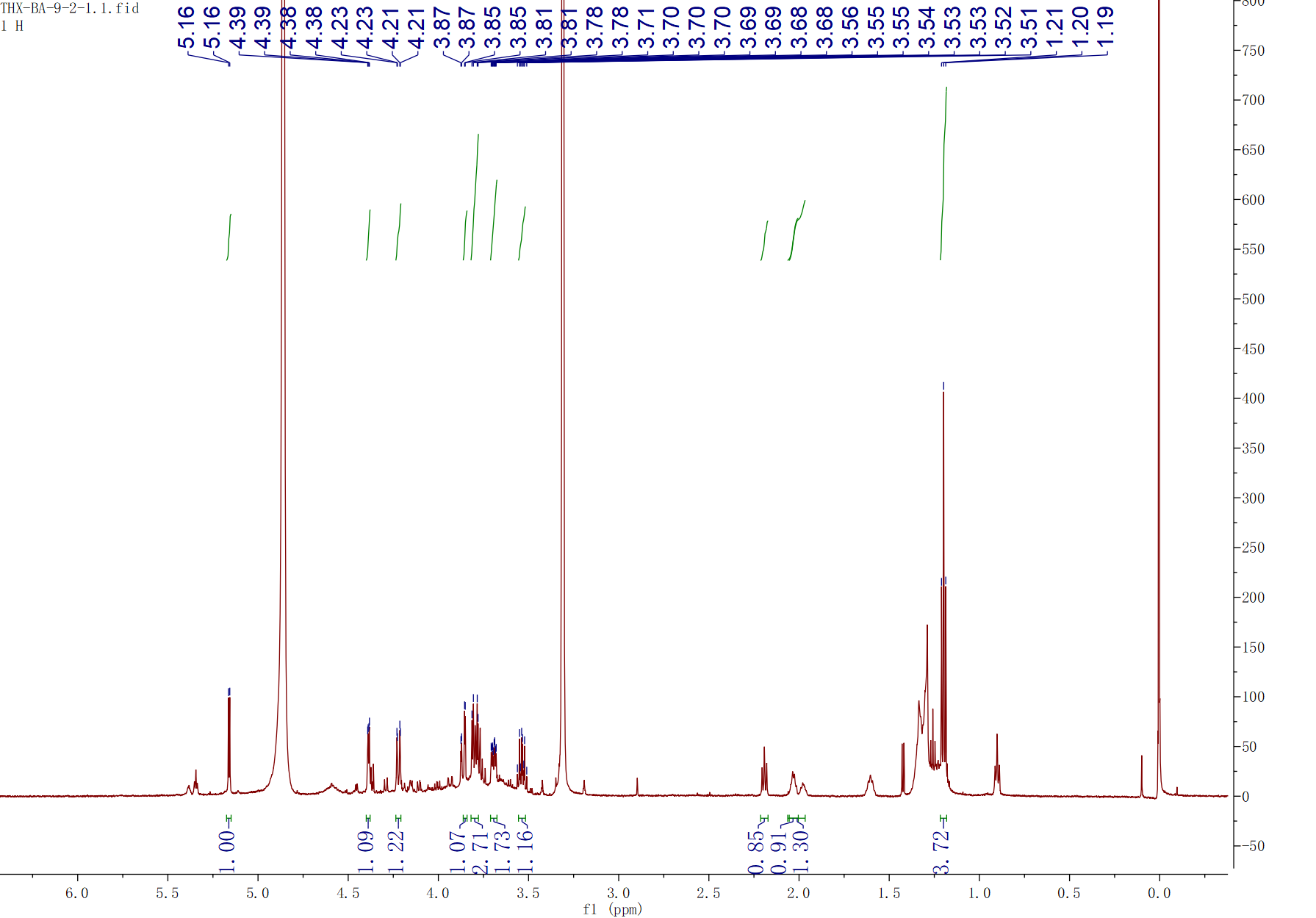


Fig. S1. ^1^H NMR spectrum of *α*-D-*ribo*-hex-3-ulopyranoside (**1**) (CD_3_OD, 600 MHz).

Fig. S2. ^13^C NMR spectrum of *α*-D-*ribo*-hex-3-ulopyranoside (**1**) (CD_3_OD, 151 MHz).


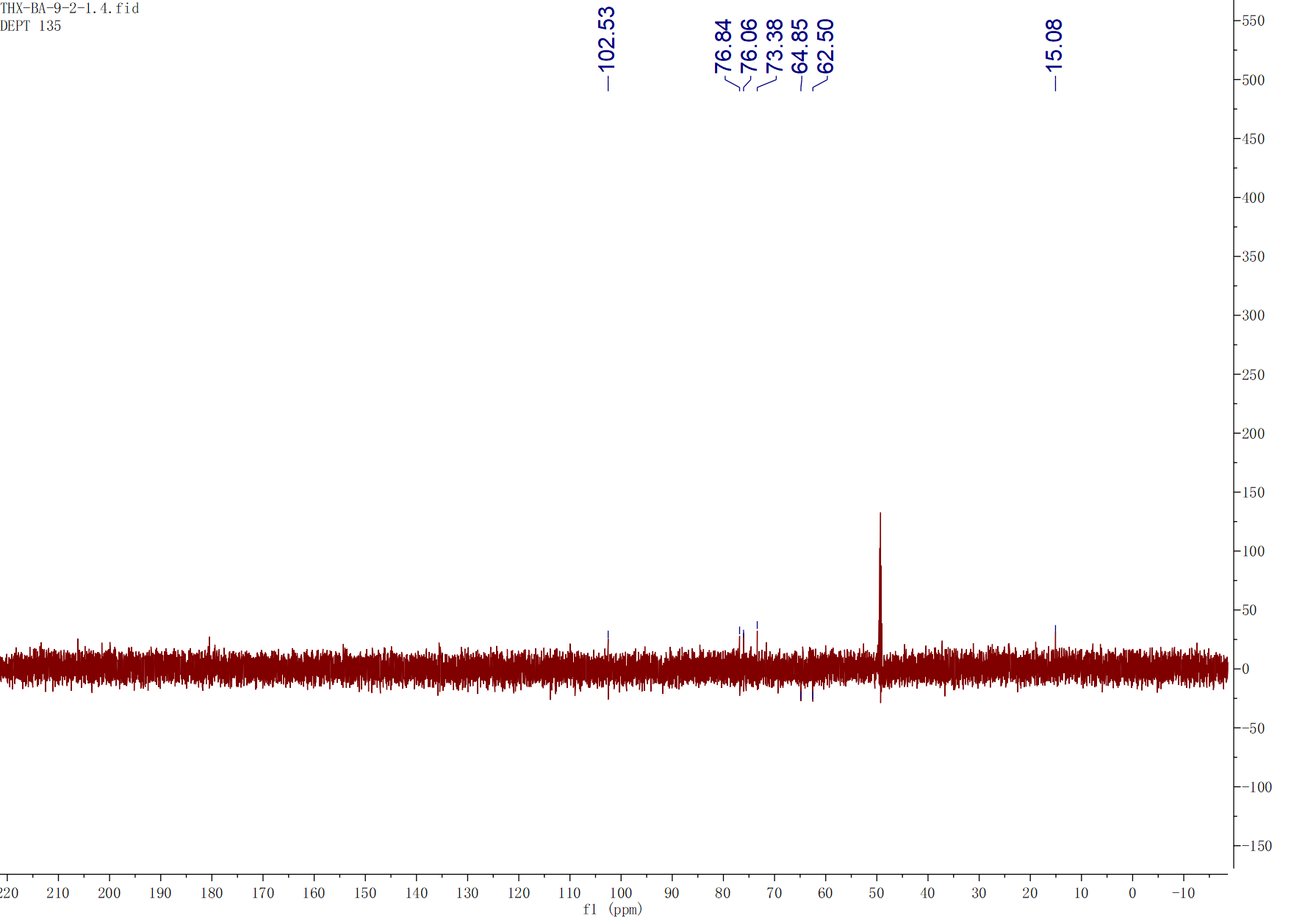


Fig. S3. DEPT 135°spectrum of *α*-D-*ribo*-hex-3-ulopyranoside (**1**).


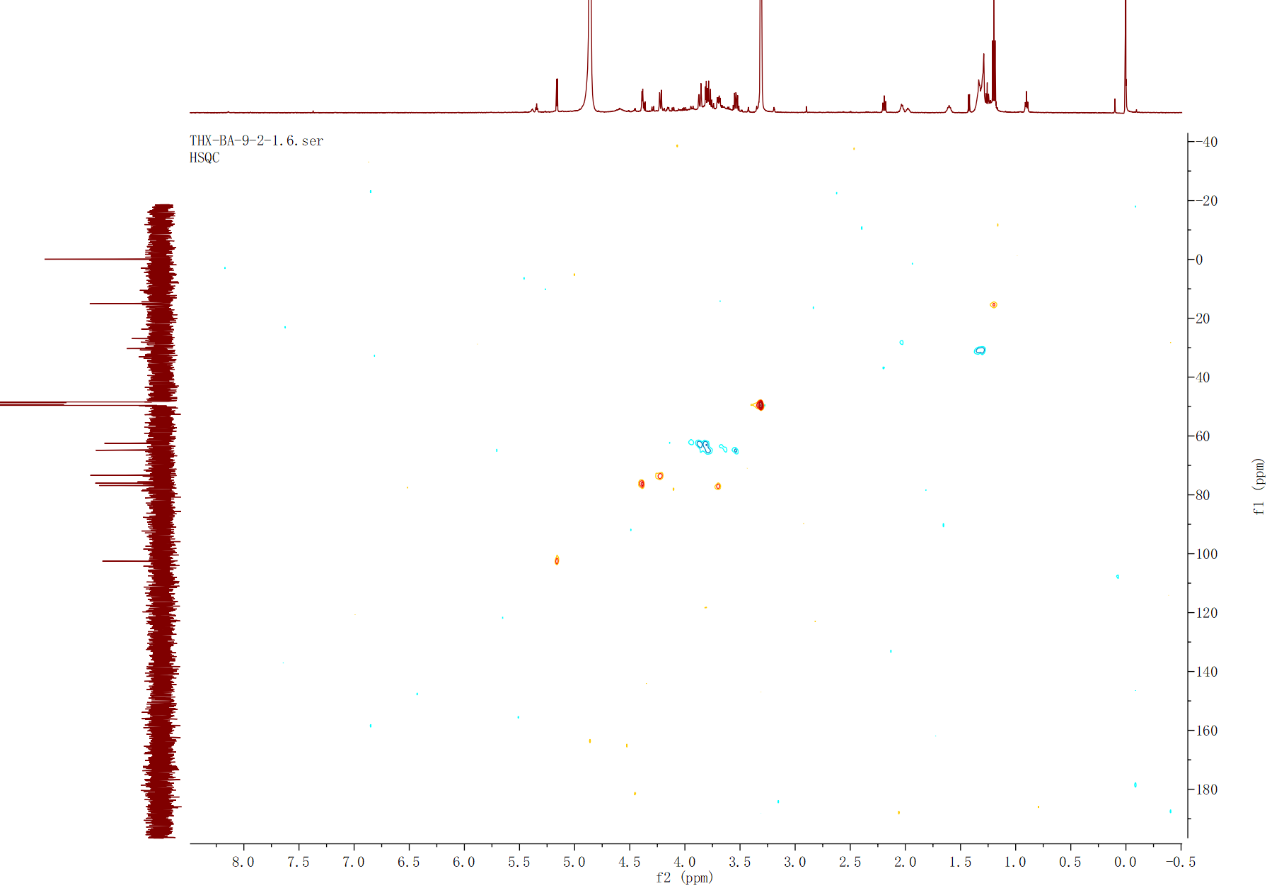


Fig. S4. HSQC spectrum of *α*-D-*ribo*-hex-3-ulopyranoside (**1**).


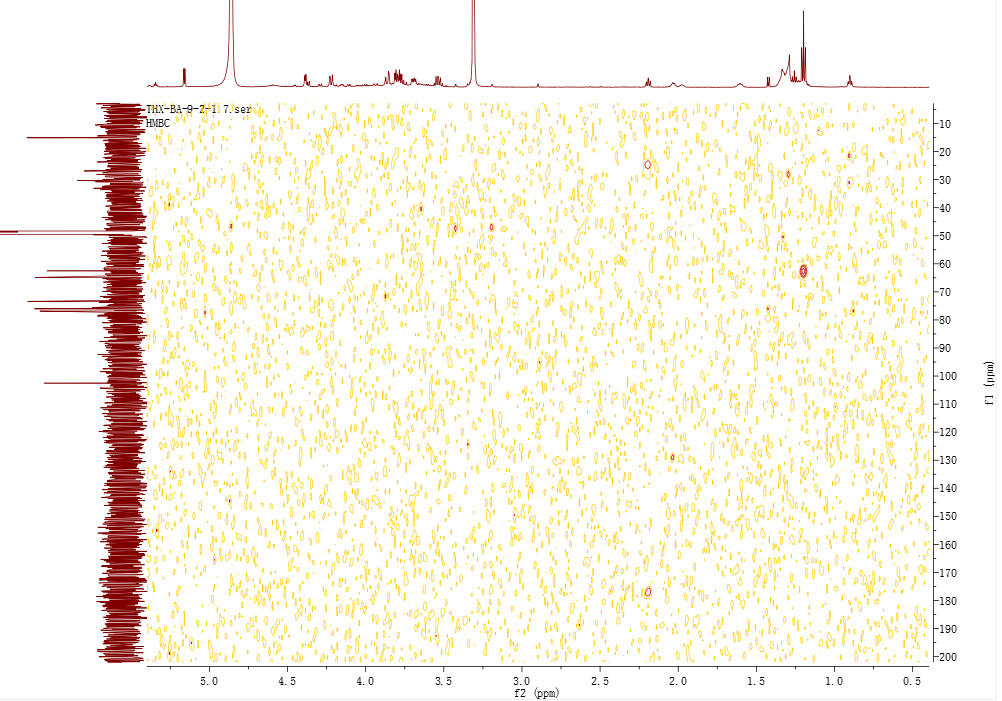


Fig. S5. HMBC spectrum of *α*-D-*ribo*-hex-3-ulopyranoside (**1**).


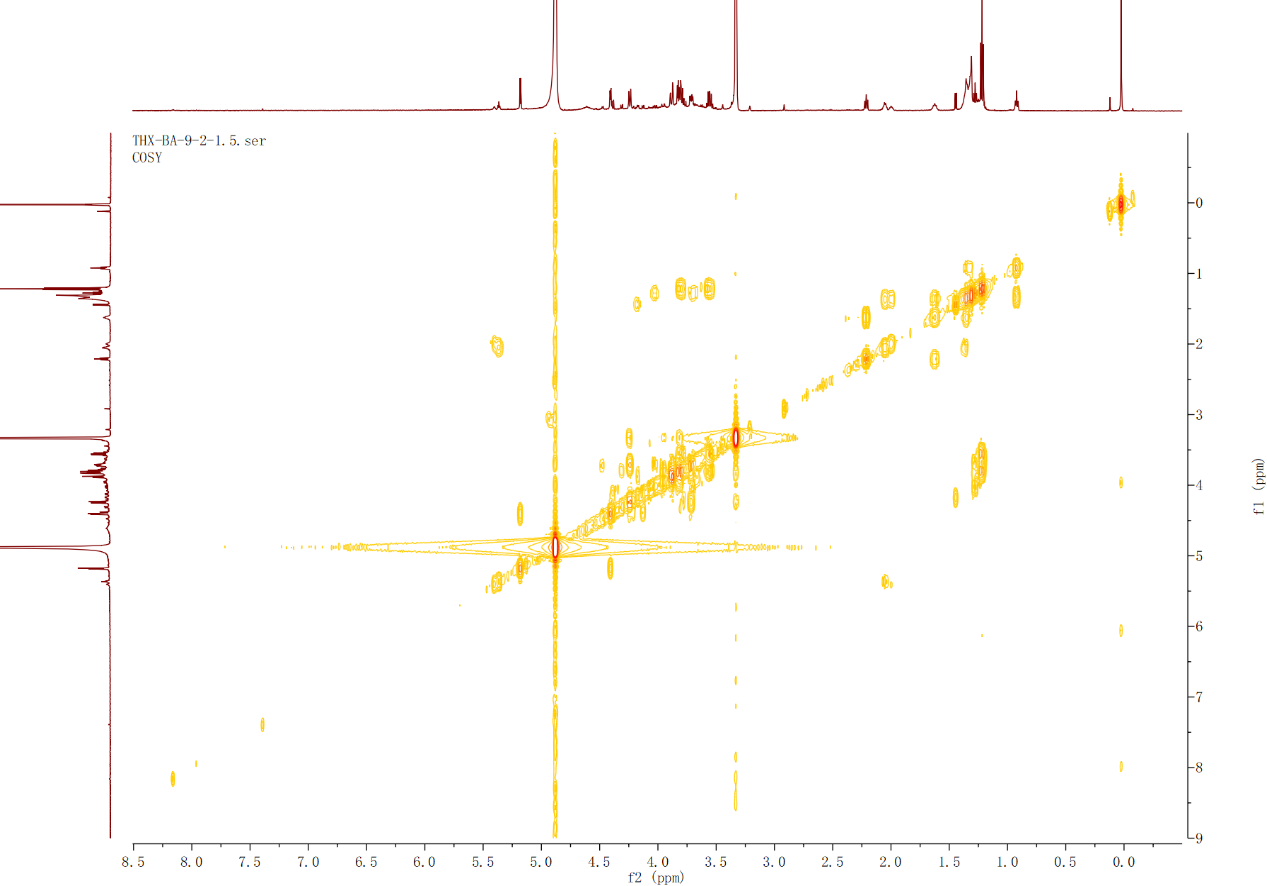


Fig. S6. ^1^H-^1^H COSY spectrum of *α*-D-*ribo*-hex-3-ulopyranoside (**1**).


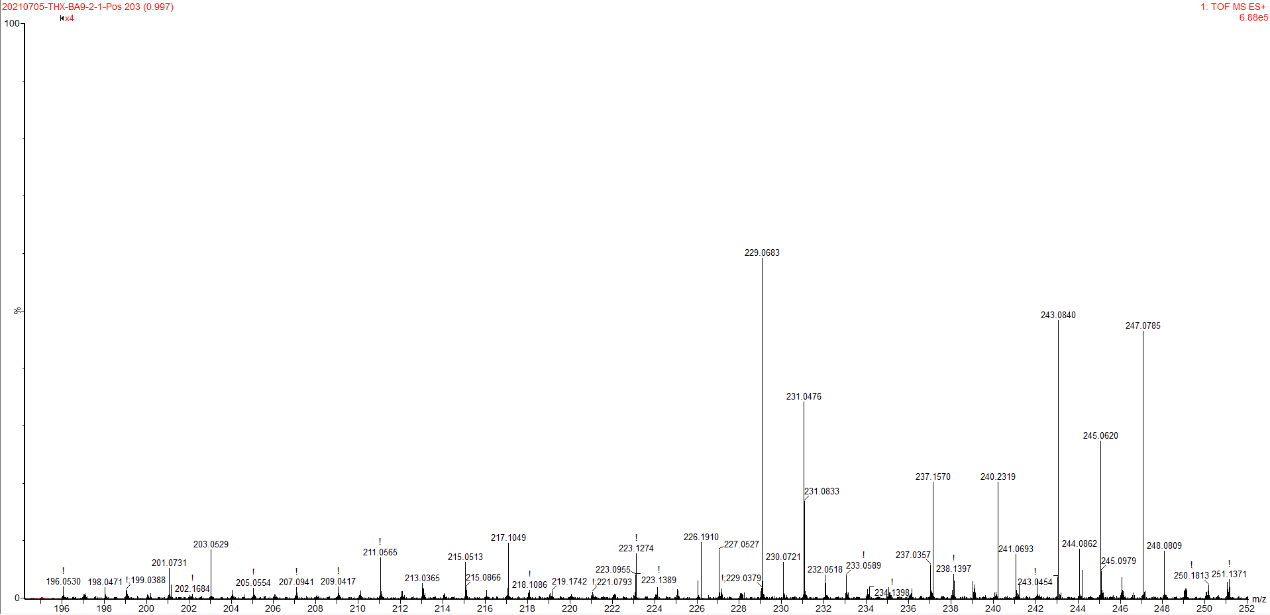


Fig. S7. HR-ESIMS spectrum of *α*-D-*ribo*-hex-3-ulopyranoside (**1**).


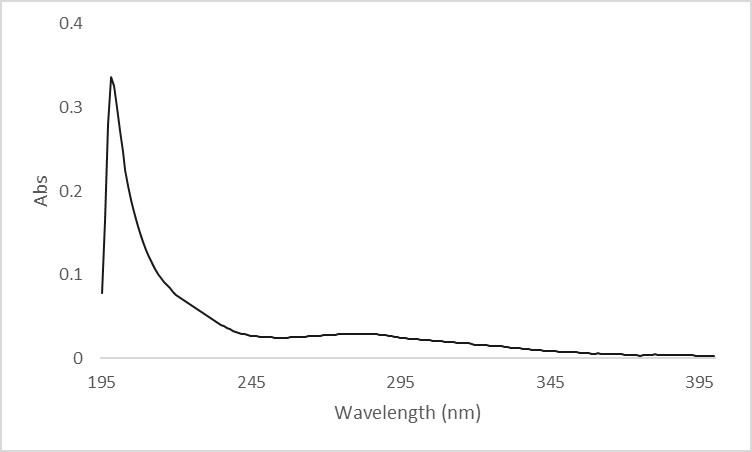


Fig. S8. UV spectrum of *α*-D-*ribo*-hex-3-ulopyranoside (**1**).


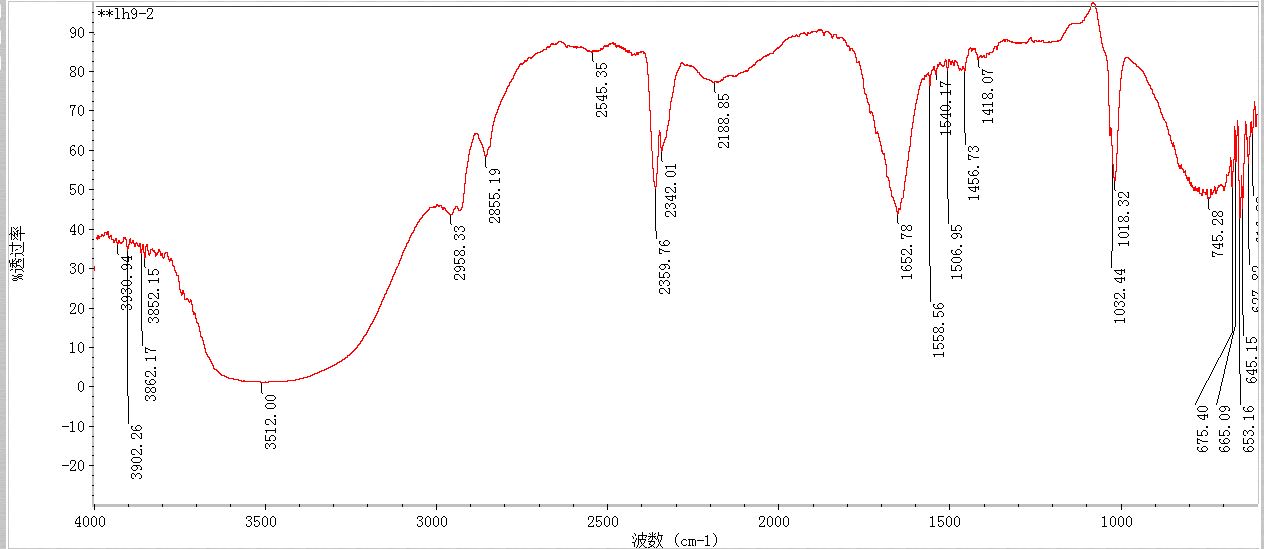


Fig. S9. IR spectrum of *α*-D-*ribo*-hex-3-ulopyranoside (**1**).


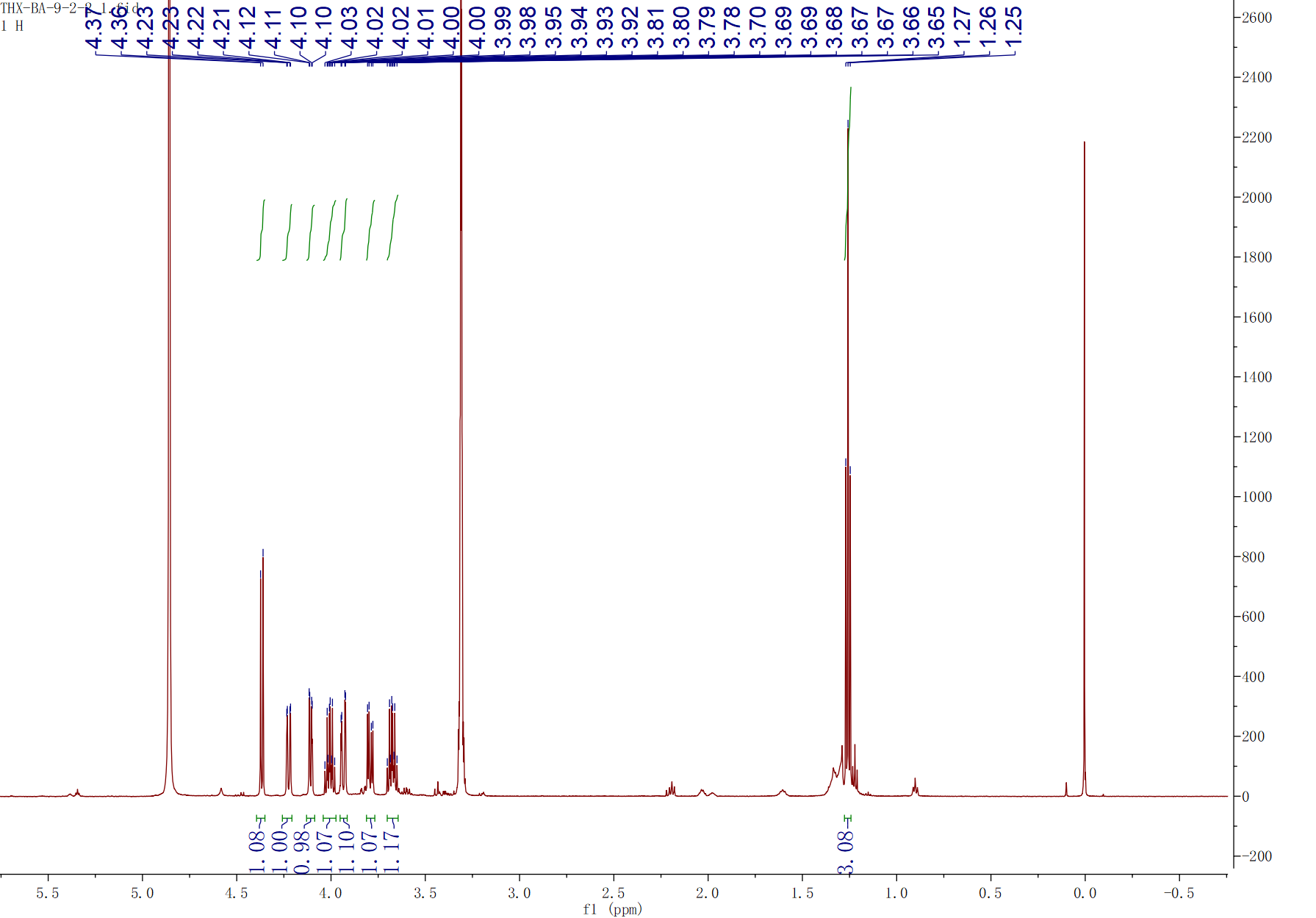


Fig. S10. ^1^H NMR spectrum of *β*-D-*ribo*-hex-3-ulopyranoside (**2**) (CD_3_OD, 600 MHz).


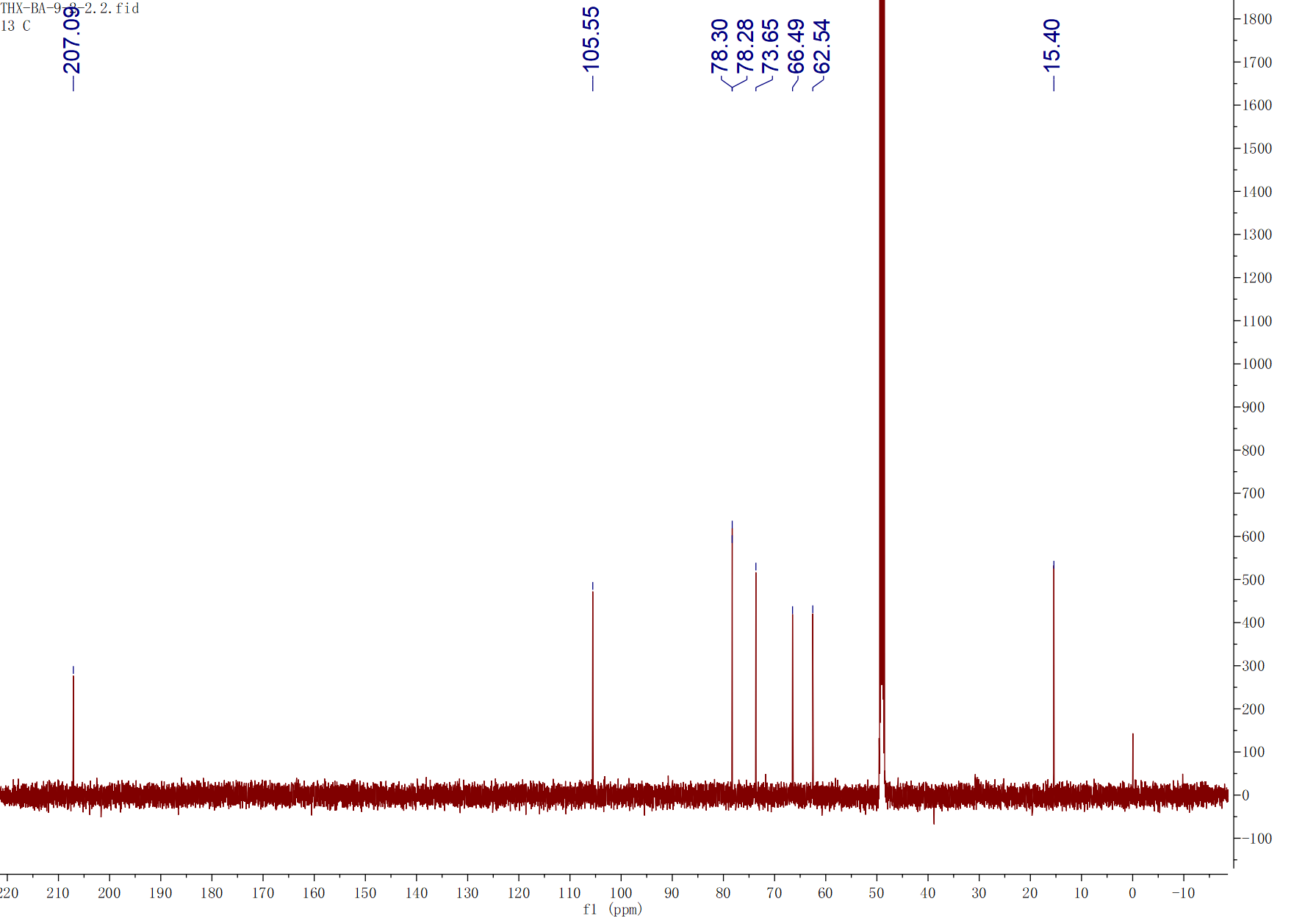


Fig. S11. ^13^C NMR spectrum of *β*-D-*ribo*-hex-3-ulopyranoside (**2**) (CD_3_OD, 151 MHz).


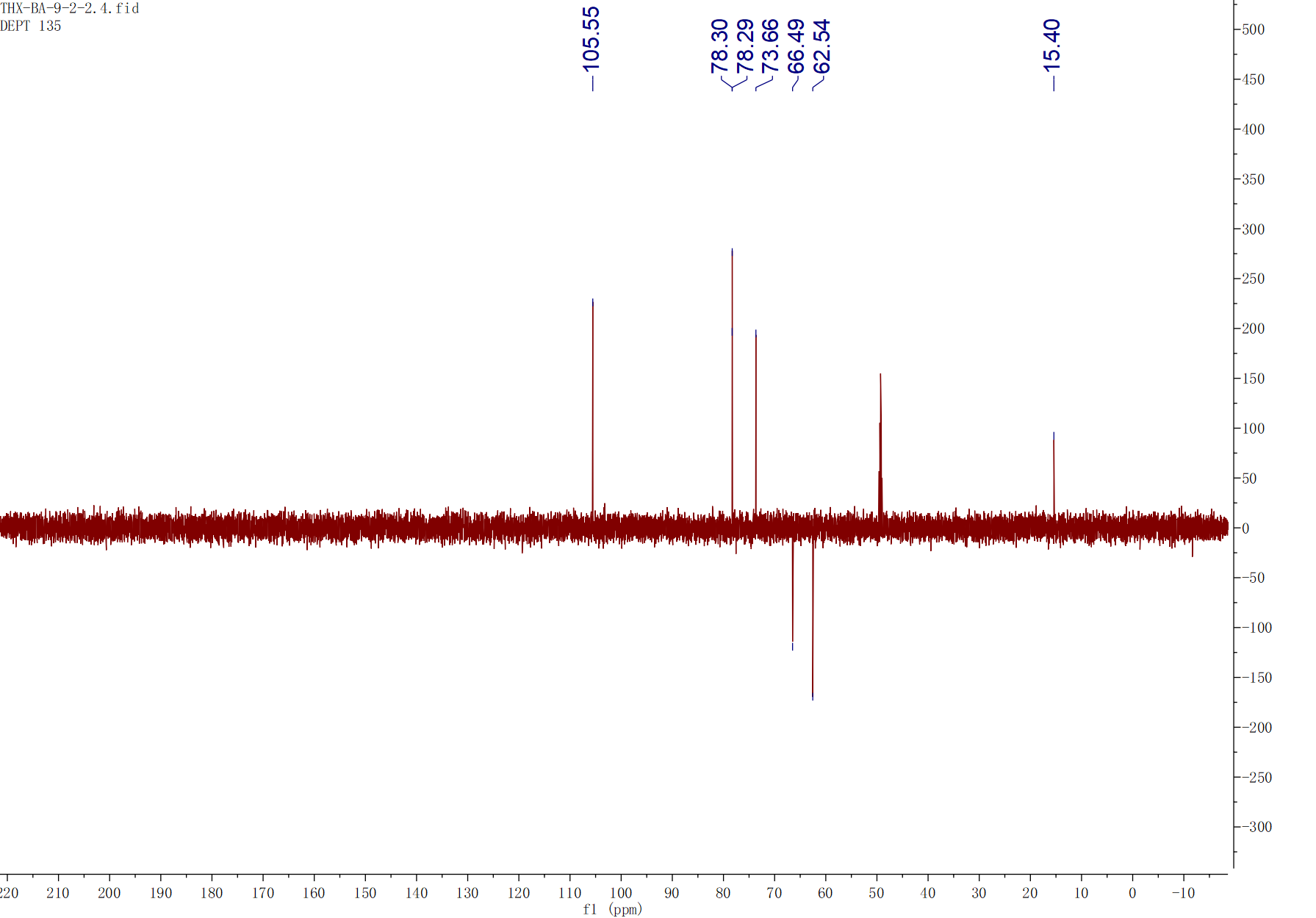


Fig. S12. DEPT 135° spectrum of *β*-D-*ribo*-hex-3-ulopyranoside (**2**).


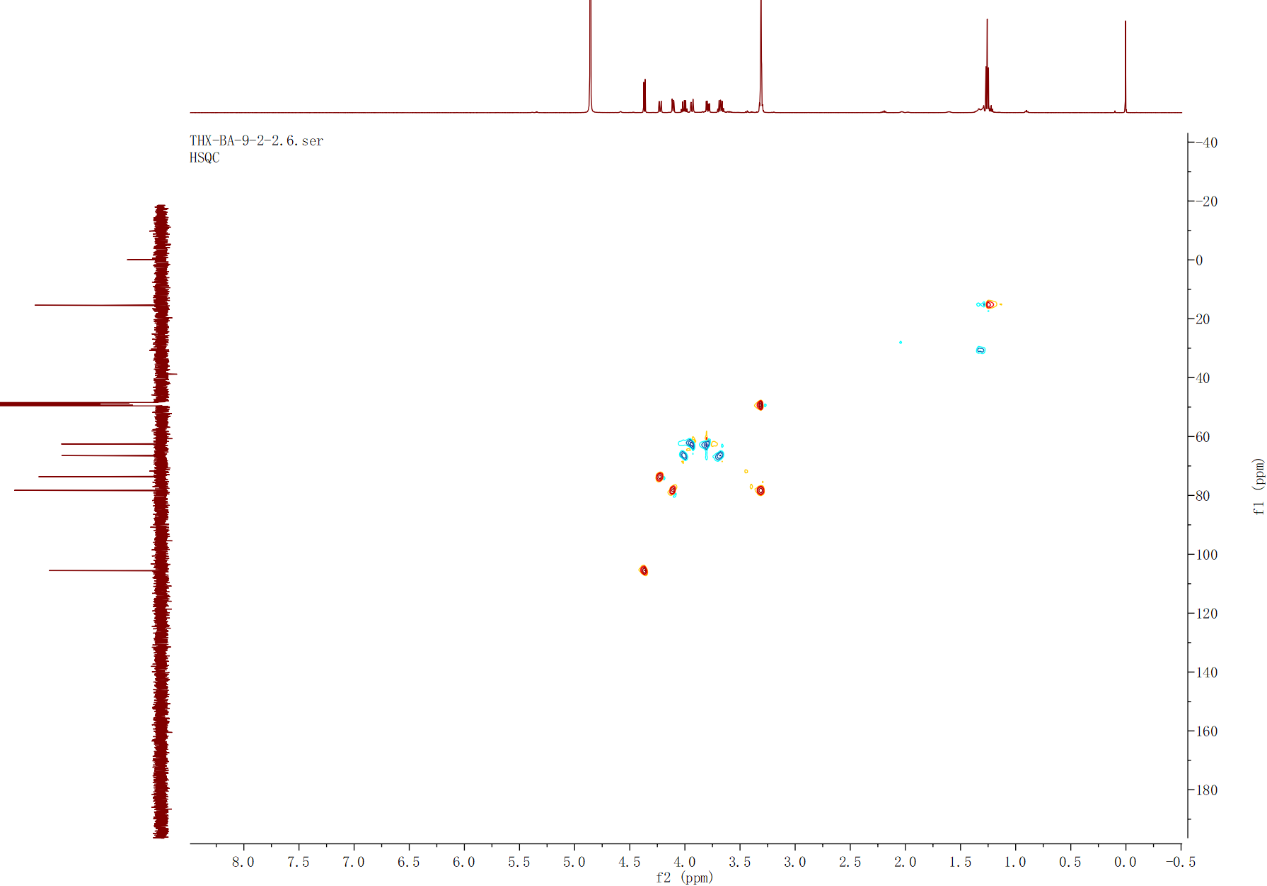


Fig. S13. HSQC spectrum of *β*-D-*ribo*-hex-3-ulopyranoside (**2**).


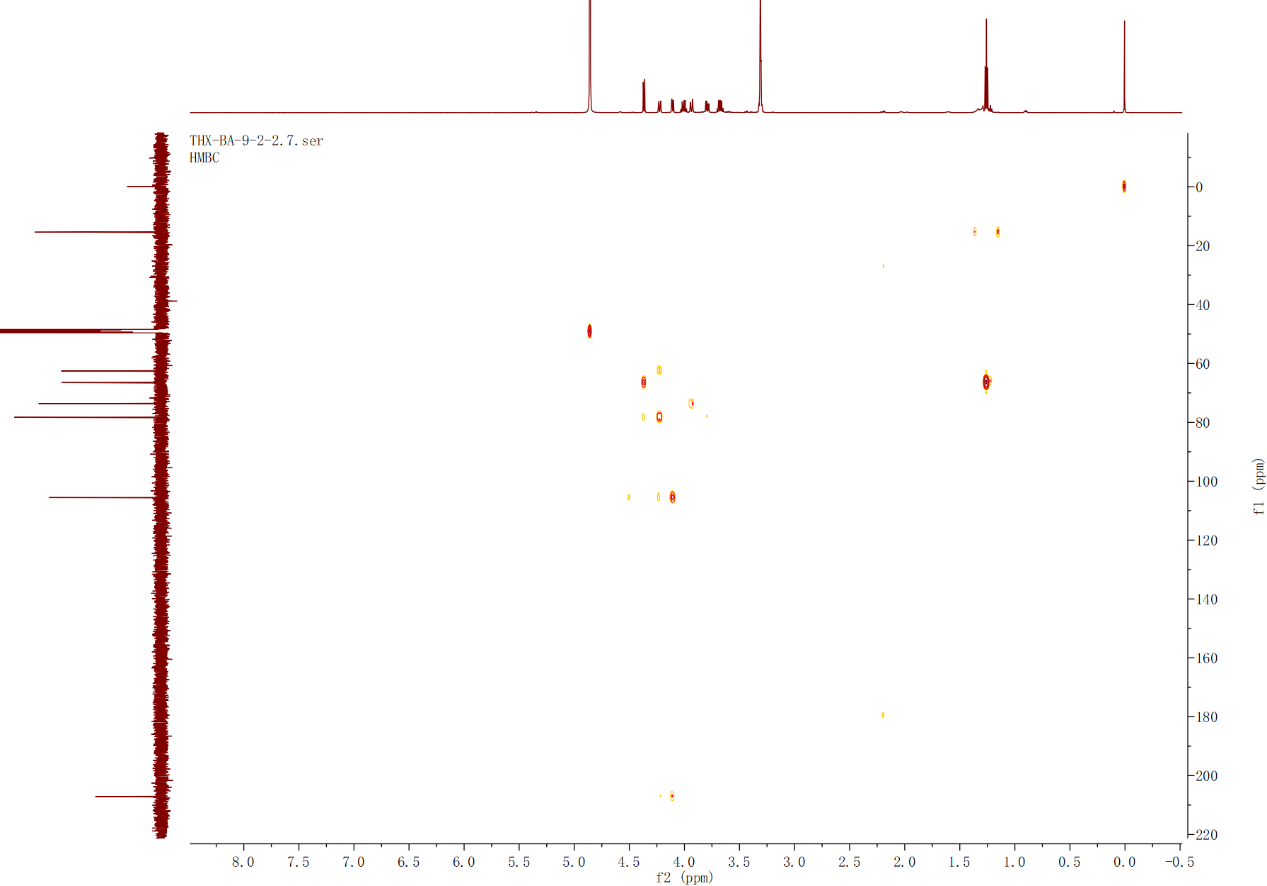


Fig. S14. HMBC spectrum of *β*-D-*ribo*-hex-3-ulopyranoside (**2**).


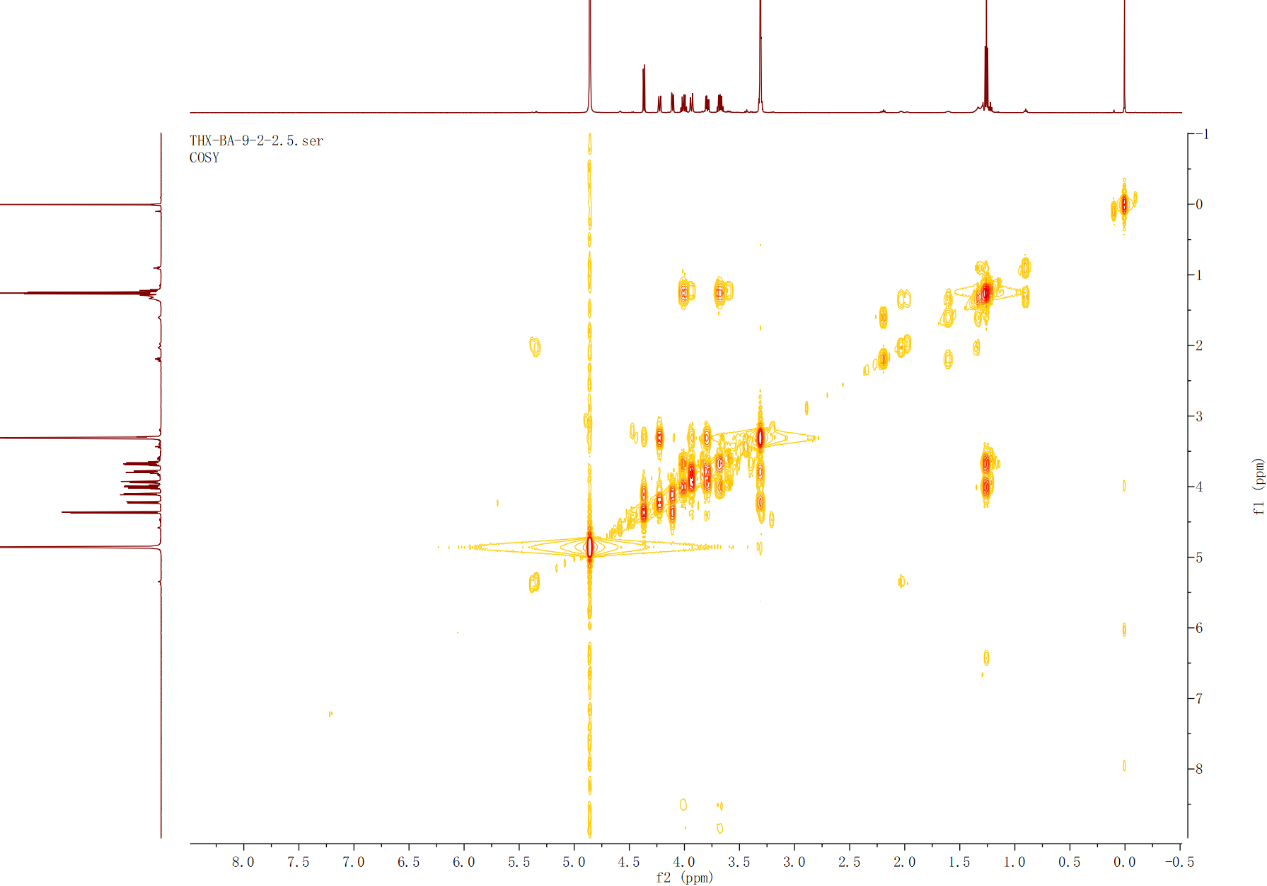


Fig. S15. ^1^H-^1^H COSY spectrum of *β*-D-*ribo*-hex-3-ulopyranoside (**2**).


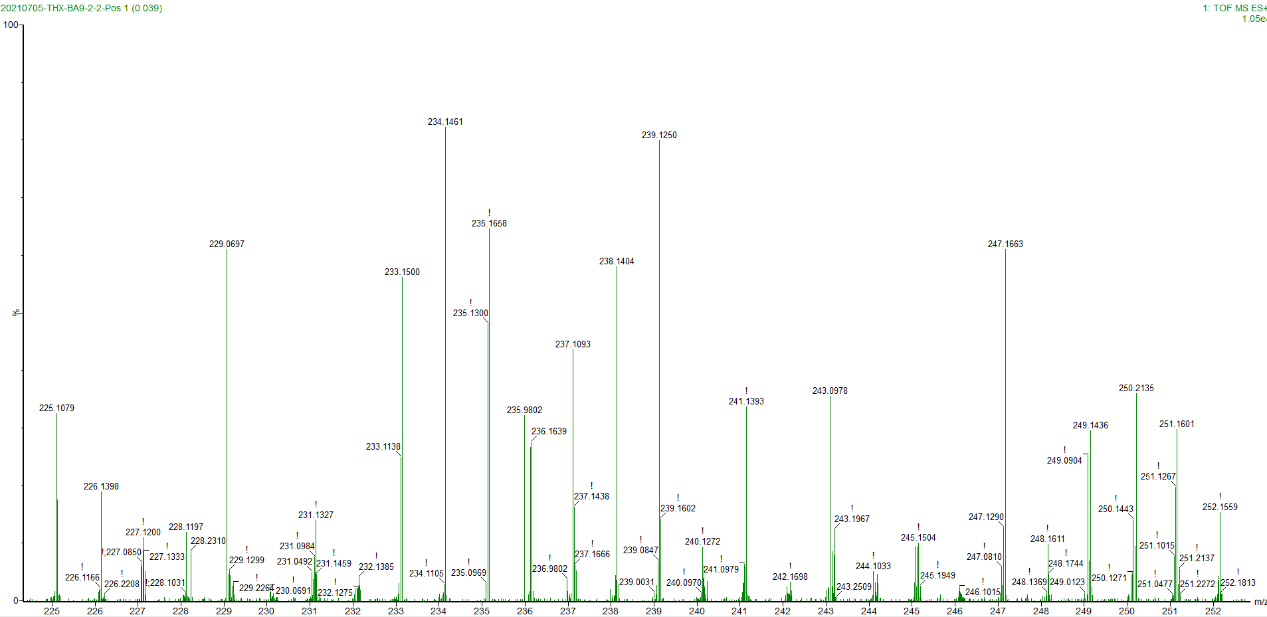


Fig. S16. HR-ESIMS spectrum of *β*-D-*ribo*-hex-3-ulopyranoside (**2**).


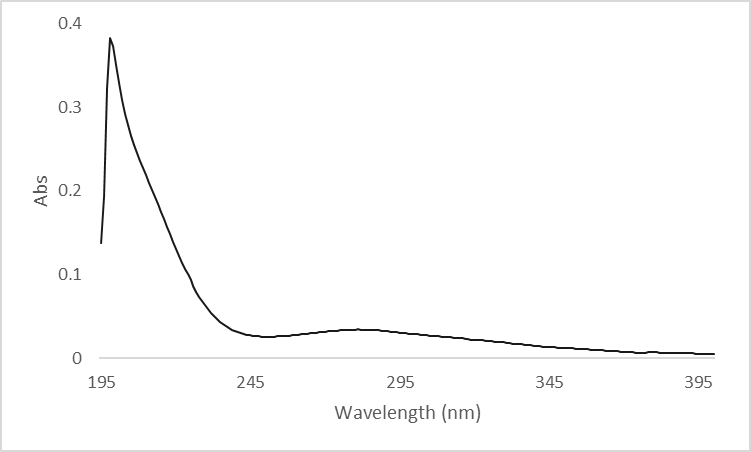


Fig. S17. UV spectrum of *β*-D-*ribo*-hex-3-ulopyranoside (**2**).


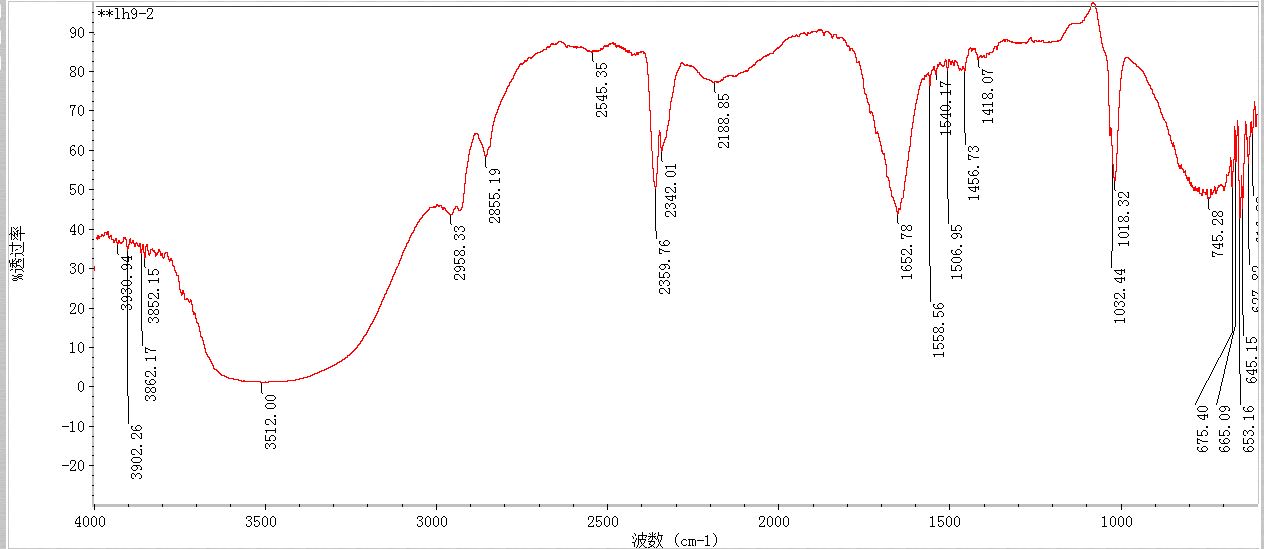


Fig. S18. IR spectrum of *β*-D-*ribo*-hex-3-ulopyranoside (**2**).
